# Interorgan amino acid interchange in propionic acidemia: the missing key to understanding its physiopathology

**DOI:** 10.1007/s00726-022-03128-6

**Published:** 2022-01-30

**Authors:** Sinziana Stanescu, Amaya Belanger-Quintana, Borja Manuel Fernandez-Felix, Pedro Ruiz-Sala, Mercedes del Valle, Fernando Garcia, Francisco Arrieta, Mercedes Martinez-Pardo

**Affiliations:** 1 Unidad de Enfermedades Metabólicas, Hospital Universitario Ramón y Cajal, IRYCIS, Crta de Colmenar Viejo, km 9,100, PC 28034, Madrid, Spain; 2 Unidad de Bioestadistica Clinica, Instituto Ramon y Cajal de Investigacion Sanitaria. Hospital Universitario Ramón y Cajal, Crta de Colmenar Viejo, km 9,100, PC 28034, Madrid, Spain; 3 Centro de Diagnóstico de Enfermedades Moleculares, Centro de Biología Molecular, Universidad Autónoma de Madrid, CIBERER, IdiPAZ, C/Francisco Tomás y Valiente, 7, PC 28049, Madrid, Spain; 4 Unidad de Enfermedades Metabólicas, Hospital Universitario Ramón y Cajal, IRYCIS, CIBER-OBN, Crta de Colmenar Viejo, km 9,100, PC 28034, Madrid, Spain; 5 Unidad de Enfermedades Metabólicas, Hospital Universitario Ramón y Cajal, Crta de Colmenar Viejo, km 9,100, PC 28034, Madrid, Spain

**Keywords:** Propionic acidemia, Anaplerosis, Hyperammonemia, Glutamine, Alanine, Urea cycle

## Abstract

**Background:**

Propionic acidemia is an inborn error of metabolism caused by a deficiency in the mitochondrial enzyme propionyl-CoA carboxylase that converts the propionyl CoA to methyl malonyl CoA. This leads to profound changes in distinct metabolic pathways, including the urea cycle, with consequences in ammonia detoxification. The implication of the tricarboxylic acid cycle is less well known, but its repercussions could explain both some of the acute and long-term symptoms of this disease.

**Materials and methods:**

The present observational study investigates the amino acid profiles of patients with propionic acidemia being monitored at the *Hospital Ramón y Cajal* (Madrid, Spain), between January 2015 and September 2017, comparing periods of metabolic stability with those of decompensation with ketosis and/or hyperammonemia.

**Results:**

The concentrations of 19 amino acids were determined in 188 samples provided by 10 patients. We identified 40 metabolic decompensation episodes (22 only with ketosis and 18 with hyperammonemia). Plasma glutamine and alanine levels were reduced during these metabolic crises, probably indicating deficiency of anaplerosis (*p* < 0.001 for both alanine and glutamine). Hypocitrulllinemia and hypoprolinemia were also detected during hyperammonemia (*p* < 0.001 and 0.03, respectively).

**Conclusions:**

The amino acid profile detected during decompensation episodes suggests deficient anaplerosis from propionyl-CoA and its precursors, with implications in other metabolic pathways like synthesis of urea cycle amino acids and ammonia detoxification.

## Introduction

Propionic acidemia (PA; MIM #606054) is an inherited autosomic recessive metabolic disease caused by a deficiency in the propionyl-CoA carboxylase, a mitochondrial enzyme that transforms propionyl-CoA into methyl malonyl-CoA which later enters the tricarboxylic acid (TCA) cycle after its conversion to succinyl-CoA by the methyl malonyl-CoA mutase. The sources of propionyl-CoA include the propiogenic amino acids valine (Val), methionine (Met), isoleucine (Ile) and threonine (Thr), odd-numbered long chain fatty acids (OLCFAs), the side chains of cholesterol, and propionate produced by intestinal bacteria performing fermentation (Baumgartner et al. 2014; Wongkittichote et al. 2017).

PA can present at any age, but most patients begin to experience symptoms as neonates, with episodes of hyperammonemia, metabolic acidosis with an increased anion gap, hyperlactacidemia, hypo/hyperglycemia, rhabdomyolysis and/or abnormal liver function. The resulting acute multiorgan failure and increased mortality rate or the long-term brain damage are usually attributed to the presence of hyperammonemia (Baumgartner et al. 2014); Savy et al. 2018; Haijes et al. 2019).

Several mechanisms have been postulated to explain elevated ammonia levels during metabolic decompensation in PA. It has been proposed that the main factor is that the accumulation of propionyl-CoA inhibits the enzyme *N*-acetylglutamate synthetase (NAGS) which catalyzes the formation of *N*-acetyl glutamate (NAG) needed to activate carbamoyl phosphate synthetase 1 (CPS1), a key mitochondrial enzyme in starting the urea cycle (Dercksen et al. 2014). Some evidence also suggests the amino acid substrates of the urea cycle, especially citrulline (Cit), ornithine (Orn) and arginine (Arg) are reduced in patients with PA (Scholl-Bürgi et al. 2012) (Scholl-Bürgi et al. 2010; Haijes et al. 2020).

Most efforts in the treatment of PA up to now have been aimed to avoid hyperammonemia episodes, which are considered the marker of a good or bad metabolic control. Traditionally, a low-protein diet with restriction of propiogenic amino acids was the main therapy. Nowadays, the use of a *N*-acetylglutamate analog or hepatic transplantation are being proposed as useful therapies to minimize the number of decompensations (Forny et al. 2021). Improvements in the diagnosis and treatment of PA are not always successful in avoiding decompensations and poor neurological outcomes. For this reason, some countries are reticent to begin or have stopped neonatal screening for PA (Forny et al. 2021). Better treatment has allowed more patients to survive into adulthood, but older patients frequently experience acute and long-term complications besides the ones related to brain damage, such as cardiomyopathy, ictus-like episodes, pancreatitis, anemia/pancytopenia, and atrophy of the optic nerve (Haijes et al. 2019). It is currently impossible to predict in which patients they will appear as they can happen both in cases with and without what is considered an adequate metabolic control. The pathophysiology of these complications is not well understood, but it is clear that they cannot all be explained by the deleterious effect of high ammonia levels.

The toxic effect of other metabolites besides ammonia can be an explanation. Especially methyl citrate has been especially related neurological complications (Haijes et al. 2019). A secondary dysfunction of energetic metabolic pathways such as the tricarboxylic cycle (TCA) or the mitochondrial respiratory chain are also pathogenic mechanisms under investigation (Longo et al. 2017; Haijes et al. 2019). The propionate pathway feeds the TCA cycle through the propionyl CoA, an important anaplerotic molecule (Brunenrgaber and Roe 2006). For propionyl-CoA to enter the TCA cycle as succinyl-CoA, both propionyl-CoA carboxylase and methyl malonyl-CoA mutase must be unimpaired. We understand that in PA patients this anaplerotic pathway is severely disturbed due to the lack of propionyl-CoA carboxylase and the consequently deficiency of succinyl-CoA. Moreover, the excess of propionyl-CoA sequestrates the oxaloacetate to form methyl citrate, and further depletes the TCA cycle (Brunenrgaber and Roe 2006; Filipowicz et al. 2006). These mechanisms contribute to a deficient flux of Krebs cycle intermediates and decreased availability of its substrates such as citrate or α-ketoglutarate (Filipowicz et al. 2006; Longo et al. 2017).

The aim of the present work was to compare the plasma amino acid profiles of our patients with PA during episodes of metabolic stability and periods of decompensation in the hope of gaining a better understanding of the biochemical alterations that occur in patients with PA which might help explain the appearance of acute and long-term complications, and subsequently improve treatment and ameliorate patient survival and quality of life.

## Materials and methods

This prospective observational study investigates the aminograms of patients with PA being monitored at a metabolic reference center (Hospital Ramón y Cajal, Madrid, Spain) between January 2015 and September 2017.

During periods of metabolic stability, blood was collected coinciding with routine hospital appointments, after a minimum 6 h of fasting and without having consumed any carnitine in the previous 8 h. Samples were also collected during periods of decompensation before the start of any dietetic or pharmacological treatment. Metabolic decompensation was defined as hyperammonemia (plasma NH_4_ > 60 μmol/L) and/or the significant presence of ketone bodies in the urine as determined by urine dipsticks (≥ + +). Plasma amino acid analysis was carried out on a Biochrom 30 Analyzer by cation exchange liquid chromatography process after protein precipitation with sulfosalicylic acid. The amino acids react post-column with ninhydrin, giving rise to compounds evaluated on-line by spectrophotometry at different wavelengths. All laboratory measurements were completed in our ERNDIM approved, reference laboratory (CEDEM, Centro de Diagnóstico de Enfermedades Moleculares, Universidad Autónoma, Madrid, Spain).

The study protocol was approved by the hospital’s Ethics Committee and all patients, or their legal guardians signed an informed consent prior to their inclusion.

The means ± standard deviation, medians and 25th and 75th percentiles for plasma amino acids under metabolic stability and decompensation conditions were determined. Comparisons were made between levels obtained during periods of metabolic stability with those of decompensation with hyperammonemia or ketosis. Since repeated measures were made for each patient, the association between amino acid status and metabolic condition was examined in univariate fashion using multilevel linear regression. We considered statistically significant for a *p*-value < 0.05. All analyses were performed using Stata software version 16.

## Results

The blood concentrations of 19 amino acids were determined in 188 samples provided by 10 patients aged 5–38 years. Table 1 shows the clinical and demographic features of the patients. Samples corresponded to 148 periods of metabolic stability, and 40 decompensation episodes (22 with only ketosis and 18 also had hyperammonemia). Differences by age or sex where not included in the analyses, due to the small sample of patients. Instead, we prioritized the unified criteria of blood specimen collection (minimum 6 h of fasting in case of stability or before any dietary or pharmacological intervention during acute crisis), in order to achieve metabolically homogenous patients.

**Table 1:** Demographic and clinical data of 10 PA patients included in the study

	Sex	Current age (years)	Age at diagnosis	Genetics	Clinical course (long term complications)
**1**	M	Died (5)	Neonatal	PCCA gene p.Leu470Arg/p.Leu470Arg	Severe neuromotor delay choreoathetosis, basal ganglia involvement, leukopenia, frequent infections, dilated cardiomyopathy, pancreatitis Severe persistent anemia
**2**	M	9	Neonatal screening	PCCB p.Asn536Asp/p.Asn536Asp	Autism
**3**	M	12	6 months	PCCA gene p.Gly477fs*9/ p.Cys616_Val633del	Sever neuromotor delay
**4**	M	12	Neonatal	PCCB gene p.Gly407Argfs*14/ p. Arg410Trp	Severe neuromotor delay, leukopenia Severe persistent anemia
**5**	F	13	6 months	PCCB gene p.Arg512Cys/p.Gly255Ser	Neuromotor delay, epilepsy, pancreatitis, myositis
**6**	F	15	4 months	PCCA gene p.Gly477fs*9/ p.Cys616_Val633del	Pancreatitis
**7**	F	27	4 months	PCCB gene p.Gly407Argfs*14/p.Glu168Lys	–
**8**	F	32	Neonatal	PCCB gene p.Gly407Argfs*14/ p.Gly407Argfs*14	Neuromotor delay, dilated cardiomyopathy
**9**	F	32	Neonatal	PCCB gene p.Gly407Argfs*14/p.Arg165Gln	Peripheric neuropathy, neuromotor delay, pancreatitis, thrombopenia Severe persistent anemia
**10**	F	38	Neonatal	PCCB p.Glu168Lys/p.? (c.183 + 3G > C)	Neuromotor delay

**Fig. 1:**
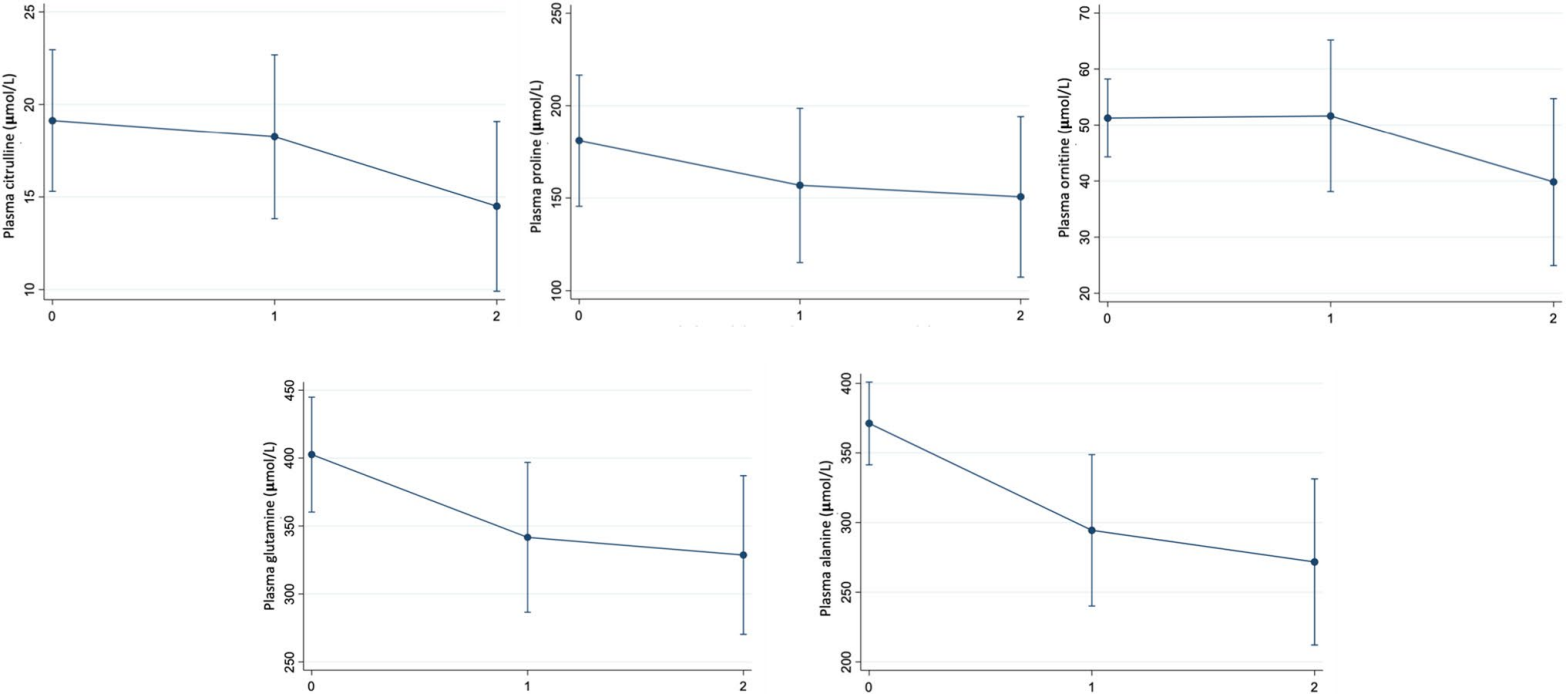
Multilevel linear regression analysis of plasma amino acid levels according to metabolic status using (0: metabolic stability, 1: decompensation with ketosis, 2: decompensation with hyperammonemia)

Plasma glutamine (Gln) was reduced during periods of decompensation (*p*-value < 0.001)*,* while no difference was seen in glutamate (Glu). The plasma alanine (Ala) concentration also was significantly lower during periods of decompensation with both hyperammonemia and ketosis (*p*-value < 0.001), see Fig. 1 and Tables 2 and 3.

**Table 2:** Descriptive analysis of plasma amino acid levels according to metabolic state (metabolic stability, decompensation with ketosis and decompensation with hyperammonemia)

	Metabolic status		
	Metabolic stability	Metabolic decompensation with ketosis	Metabolic decompensation with hyperammonemia
	*n* = 148	*n* = 22	*n* = 18
	Mean (SD) Median (p25;p75)	Mean (SD) Median (p25;p75)	Mean (SD) Median (p25;p75)
Glycine	614 (304) 572 (355; 854)	687 (308) 698 (436; 1011)	662 (276) 651 (445; 748)
Lysine	170 (59) 157 (127; 207)	174 (92) 147 (131; 191)	178 (88) 163 (108; 204)
Proline	173 (70) 158 (122; 209)	146 (74) 113 (96; 179)	134 (68) 116 (95; 138)
Alanine	364 (111) 357 (290; 421)	295 (92) 291 (233; 352)	250 (170) 241 (118; 354)
Glutamine	393 (103) 394 (317; 466)	330 (91) 304 (272; 408)	303 (94) 316 (228; 377)
Glutamate	93 (62) 83 (58; 112)	87 (58) 76 (57; 106)	92 (35) 85 (71; 126)
Serine	157 (50) 149 (127; 179)	160 (73) 140 (111; 174)	152 (49) 151 (125; 197)
Isoleucine	32 (27) 27 (20; 36)	60 (100) 26 (23; 60)	63 (99) 29 (18; 32)
Leucine	81 (46) 65 (52; 96)	113 (92) 79 (55; 119)	130 (107) 89 (40; 199)
Valine	71 (38) 68 (45; 84)	95 (78) 65 (44; 102)	112 (121) 63 (30; 97)
Methionine	15 (6) 13 (10; 18)	14 (6) 12 (10; 18)	14 (5) 13 (11; 19)
Threonine	56 (27) 51 (39; 66)	50 (22) 45 (37; 65)	60 (22) 61 (47; 77)
Aspartate	11 (6) 10 (8; 12)	12 (12) 9 (7; 12)	12 (8) 10 (6; 12)
Arginine	40 (20) 37 (27; 50)	36 (25) 32 (20; 44)	33 (18) 33 (21; 48)
Citrulline	17 (7) 17 (14; 22)	14 (6) 12 (9; 21)	9 (5) 9 (7; 12)
Ornithine	50 (28) 44 (31; 64)	49 (37) 30 (26; 62)	43 (29) 34 (26; 49)
Phenylalanine	45 (15) 45 (39; 54)	55 (36) 47 (35; 58)	46 (15) 44 (35; 54)
Tyrosine	58 (28) 49 (38; 72)	58 (32) 51 (44; 59)	50 (24) 47 (33; 55)
Tryptophan	43 (22) 41 (29; 57)	42 (23) 39 (26; 52)	33 (23) 29 (18; 37)
Fischer’s ratio	1.8 (0.6) 1.8 (1.4; 2.2)	2.4 (1.4) 1.8 (1.6; 2.9)	3 (2.5) 1.9 (1.5; 2.7)
Glutamine + Glutamate	486 (113) 478 (404; 565)	416 (92) 370 (339; 515)	396 (105) 388.5 (323; 477)
Glutamate/Glutamine	0.27 (0.38) 0.21 (0.14; 0.30)	0.3 (0.29) 0.23 (0.14; 0.29)	0.32 (0.12) 0.368 (0.21; 0.42)

**Table 3:** Analysis of plasma amino acid levels according to metabolic status using multilevel linear regression

	Metabolic decompensation with ketosis		Metabolic decompensation with hyperammonemia	
	Coefficient (95% CI)	*p*-value	Coefficient (95% CI)	*p*-value
Glycine	36.5 (– 75.4; 148.5)	0.52	– 26.3 (– 149.7; 97.2)	0.67
Lysine	0.5 (– 27.1; 28.1)	0.97	– 10.6 (– 41.0; 19.8)	0.49
Proline	– 24.1 (– 48.9; 0.6)	0.05	– 30.3 (– 57.7; – 2.9)	0.03
Alanine	– 76.7 (– 281.4; 24.9)	< 0.001	– 99.4 (– 156.4; 42.4)	< 0.001
Glutamine	– 60.9 (– 100.8; 21.0)	< 0.001	– 73.9 (– 117.9; – 29.9)	< 0.001
Glutamate	– 19.7 (– 45; 5.6)	0.12	– 11.7 (– 39.7; 16.1)	0.40
Serine	– 1.7 (– 25.2; 21.8)	0.88	– 17.3 (– 43.3; 8.5)	0.18
Isoleucine	26.3 (6.1; 46.4)	0.01	28.9 (6.7; 51.1)	0.01
Leucine	29.6 (5.8; 53.3)	0.01	44.1 (17.9; 70.4)	< 0.001
Valine	23.5 (2.2; 44.7)	0.03	50.3 (26.9; 73.7)	< 0.001
Methionine	0.05 (– 2.2; 2.3)	0.96	0.9 (– 2.3; 2.7)	0.89
Threonine	– 3.1 (– 13.5; 7.1)	0.54	10.2 (– 1.1; 21.5)	0.07
Aspartate	1.3 (– 1.9; 4.5)	0.43	0.9 (– 2.6; 4.5)	0.59
Arginine	– 5.3 (– 13.5; 2.9)	0.20	– 5.9 (– 15; 3)	0.19
Citrulline	– 0.8 (– 3.3; 1.6)	0.5	– 4.6 (– 7.3; – 1.8)	< 0.001
Ornithine	0.3 (– 12.8; 13.6)	0.95	– 11.4 (– 25.9; 3.1)	0.12
Phenylalanine	9.5 (1.1; 17.9)	0.02	– 1.5 (– 10.7; 7.7)	0.75
Tyrosine	3.7 (– 5.7; 13.2)	0.43	– 4.7 (– 15.2; 5.7)	0.37
Tryptophan	– 1.8 (– 10.5; 6.8)	0.67	– 9.3 (– 18.9; 0.0)	0.05
Fischer’s ratio	0.4 (0.0; 0.9)	0.03	1.2 (0.7; 1.7)	< 0.001
Glutamate/ Glutamine	– 0.0 (– 0.2; 0.2)	0.90	0.0 (– 0.1; 0.2)	0.75
Glutamine + Glutamate	– 81.3 (– 127.7; – 34.8)	< 0.001	– 87.2 (– 138.4; – 36)	< 0.01

An important reduction was seen in plasma citrulline (Cit) during decompensation with hyperammonemia (*p*-value < 0.001). Despite de decline of ornithine (Orn levels) during hyperammonemia, it did not reach statistical significance, see Table 2. No significant changes were seen in plasma Arg, although during the study period some patients received Arg supplements which may have interfered with the final results. A reduction in plasma proline (Pro) was also observed during the decompensation with hyperammonemia (*p*-value: 0.03), see Fig. 1 and Tables 2 and 3.

During decompensation episodes (with either hyperammonemia or ketosis), the concentrations of the branched-chain amino acids (BCAAs: leucine (Leu), valine (Val), isoleucine (Ile)) were significantly higher (see Table 2 and 3) likely indicating an increased protein catabolism. Because BCAAs disturbances, changes in Fischer ratio (ratio between BCAAs and aromatic amino acids) were observed (see Tables 2 and 3). Similarly with liver disease patients, this imbalance might have implications in neurotransmitter synthesis (Fischer et al. 1974; Kinny-Köster et al. 2016). No differences were seen, however, in the concentration of the other propiogenic amino acids Met and Thr between these different times. Nor were any differences observed for glycine (Gly) or lysine (Lys), see Tables 2 and 3.

## Discussion

Despite advances in PA treatment, patients are still affected by brain damage and have a high mortality rate during acute decompensations. Hyperammonemia is usually present during these episodes and the toxic capacity of ammonia is well known. That is why they are considered the best marker of good or bad control and most efforts in PA have been aimed to reduce these acute events. Even with advances in treatment, patients still suffer from recurrent decompensations. They also experience multiple severe long-term complications that might appear even when they achieve acceptable metabolic control or undergo liver transplantation (Forny et al. 2021; Haijes et al. 2019). It is clear that we still do not understand the physiopathology of the disease well enough to find a reliable treatment for them.

Long- term treatment of PA has been aimed to the reduction of propionic acid levels by restricting the propiogenic amino acids (Val, Ile, Met, Thr) in the diet, reducing bacterial gut production of propionic with antibiotics and enhancing its elimination with carnitine. It is currently recommended that a plasma aminogram be performed every 3–6 months as a means of examining the nutritional profile of PA patients in order to monitor their dietetic treatment (Baumgartner et al. 2014). A coincidental urinary aminogram is not considered necessary as renal function or amino acid excretion is not usually affected in these patients. Several studies have examined these blood aminograms, finding disturbances that are not always consistent and without a clear explanation to their physiopathology or their significance (Filipowicz et al. 2006; Scholl-Bürgi et al. 2010; Scholl-Bürgi et al. 2012; Zwickler et al. 2014; Haijes et al. 2020).

Glutamine (Gln) levels during PA decompensations drop significantly. This is a known occurrence, and it has been suggested that this fall in Gln during hyperammonemia, quite unlike the increase seen in urea cycle deficiency patients, might be caused by the inhibition of glutamine synthetase due to the excess presence of propionyl-CoA or the highly toxic methyl citrate (Filipowicz et al. 2006; Al-Hassnan et al. 2003; Ierardi-Curto et al. 2000). However, we also observe a drop in alanine (Ala) concentrations, which has not been previously described. Low Ala levels is also a surprising find in patients that suffer from metabolic acidosis with elevated lactate and suggests that changes in plasma Ala are not related to mitochondrial dysfunction.

**Fig. 2:**
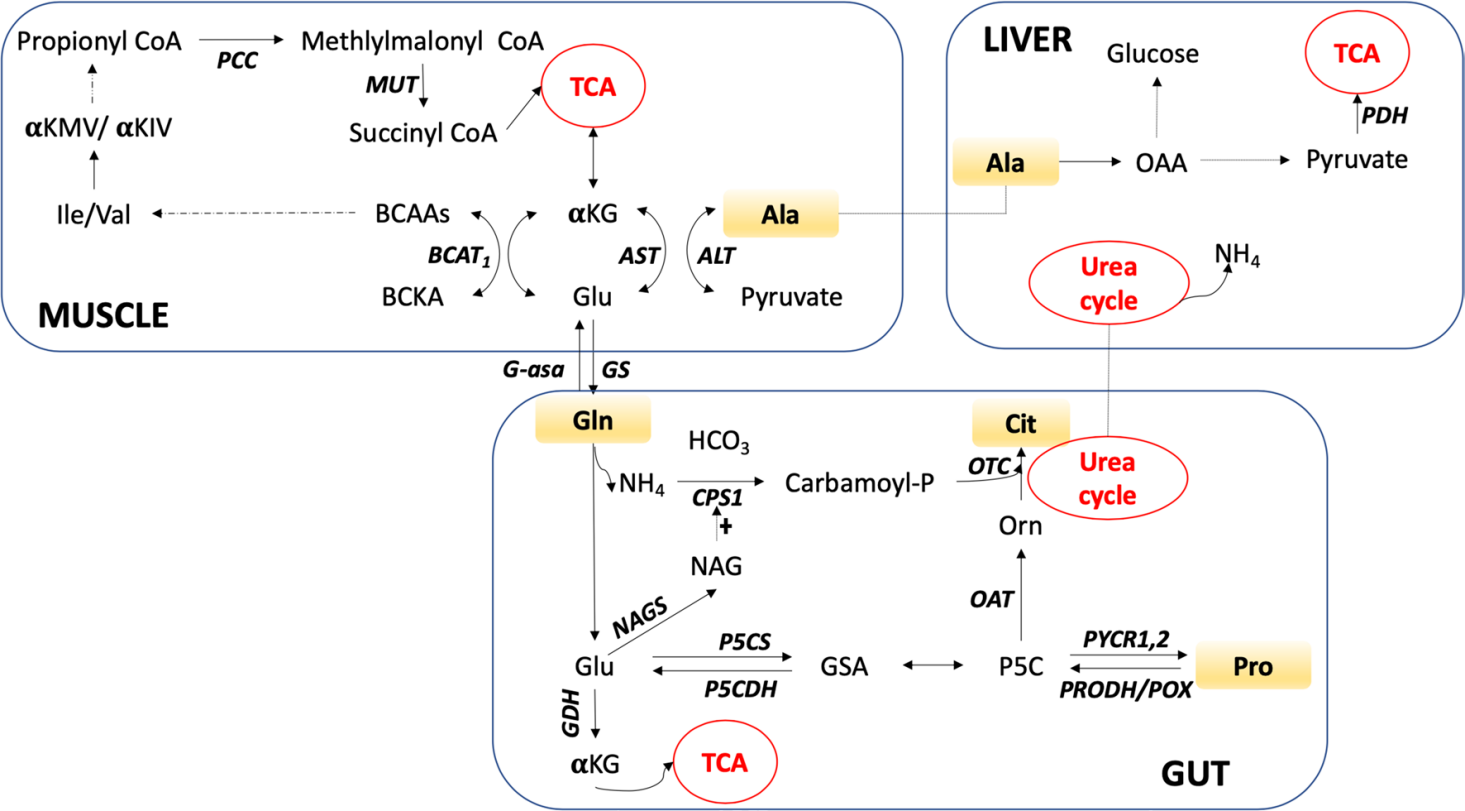
Interorgan relationship of metabolic pathways implicated in PA. *P5CS* Δ^1^-pyrroline-5-carboxylate synthetase, *G-ase* glutaminase, GS, glutamine synthetase, *NAG* *N*-acetyl-l-glutamate, *NAGS* NAG synthase, *OAT* ornithine ω-aminotransferase, *OTC* ornithine transcarbamylase, *P5CS* pyrroline-5-carboxylate synthetase, *PYCR1,2* pyrroline-5-carboxylate reductase isoforms 1 and 2, *TCA cycle* tricarboxylic acids cycle, *CPS1* carbamoyl phosphate synthetase 1, *GSA* Glutamate-5-semialdehyde, *PCC* propionyl-CoA carboxylase, *MUT* methyl malonyl- CoA mutase, *Ile* isoleucine, *Val* valine, *AST* aspartate aminotransferase, *ALT* alanine aminotransferase, *BCAT1* branched-chain amino acid aminotransferase 1, *Glu* glutamate, *Ala* alanine, *OAA* oxaloacetate, PDH: pyruvate dehydrogenase

Skeletal muscle normally releases large quantities of Gln and Ala into the plasma (up to 48% and 32% of total plasma Gln and Ala, respectively)—far more than would be expected given their own protein Gln and Ala contents (7% and 9%, respectively), indicating de novo synthesis (Wagenmakers 1998). In animal models, it has been reported that providing BCAAs to rat muscle cells led to the massive synthesis of Gln and Ala (Ruderman and Lund 1972), a finding later confirmed by other authors (Holecek et al. 2011). Certainly, it has been demonstrated that Ile and Val are the major carbon sources for Gln and Ala synthesis (Lee and Davis 1986; Chang and Goldberg 1978a, b; Brosnan and Brosnan 2006) via reactions involving Glu and α-ketoglutarate as transamination “partners” (Brosnan 2000), see Fig. 2. Glutamate and the TCA intermediate α-ketoglutarate are interconverted by reversible reactions catalyzed by glutamate dehydrogenase and aminotransferases, therefore it has been proposed that Glu/Gln pathway could be an effective anaplerotic system (Brunenrgaber and Roe 2006). From this perspective, the fall of plasma Gln and Ala during decompensation episodes might be the direct effect of a functional lack of Glu/ α-ketoglutarate.

Low Gln production by the muscle has further consequences. One of the major roles of the Gln/Glu cycle is the detoxification of ammonia, acting as a sort of transporter of this molecule to the liver in order for it to be transformed by the urea cycle (Brosnan and Brosnan 2013). The drop in Gln can be another mechanism to explain the appearance of hyperammonemia during PA decompensations.

Furthermore, Gln/Glu are involved in the synthesis of the amino acids later necessary in the urea cycle that takes place in enterocytes (Marco-Marín et al. 2020). It is well known that the intestine is a major consumer of plasma Gln (Windmueller and Spaeth 1980). In a study on inter-organ relationships in amino acid homeostasis, it was found that in healthy adults the intestinal capture of Gln is accompanied by the synthesis of Cit. The splanchnic area releases Cit, which is picked up by the kidney for the synthesis of Arg (van de Poll et al. 2007). The authors suggest a Gln–Cit–Arg pathway to exist, in which Cit/Arg are the end-products of Gln/Glu metabolism.

Recently, a new enzyme was described: ∆^1^-pyrroline-carboxylate synthetase (P5CS) is an ATP- and NADH-dependent enzyme, found in the internal mitochondrial membrane of the enterocytes and is involved in the de novo synthesis of Cit, Orn and Pro from Gln/Glu. P5CS deficiency leads to a rare disease with paradoxical pre-prandial hyperammonemia and psychomotor developmental delay, together with a peculiar metabolic profile involving hypoornithinemia, hypoprolinemia, hypocitrullinemia and hypoargininemia (Baumgartner et al. 2000). Indeed, the mitochondria of enterocytes has a developed complete enzymatic machinery—P5CS, *N*-acetyl-l-glutamate synthase (NAGS), ornithine ω-aminotransferase (OAT), and carbamoyl phosphate synthetase 1 (CPS1)—connecting Gln/Glu to the urea cycle (Marco-Marín et al. 2020; Martinelli et al. 2012), see Fig. 2. This indicates that enterocytes have a central role in the de novo synthesis of urea cycle amino acids. It has been proposed that pyrroline 5-carboxylate (P5C)/Pro couple is an obligate intermediate in the metabolic interconversions between the TCA cycle and urea cycle (Phang 2019; Wu 2009). The end metabolic product of P5C/Pro cycle are Cit/Arg on the one hand and Glu/aKG on the other, proving that Pro catabolism feeds both the urea cycle and the TCA cycle (Adams and Frank 1980; Phang 2019). From this perspective, the Gln/Glu pathway of synthesis of Orn/Cit/Arg, crossing through P5C/Pro, bridges the BCAAs muscular metabolism with ammonia detoxification and Krebs cycle.

We believe that the drop in Gln we observe not only indicates a deficient anaplerosis and reduced ammonia transport but also a dysfunction in the urea cycle due to a disturbed production of its intermediary amino acids by the enterocyte. This hypothesis is supported by the observation that our patients have a similar amino acid profile as those with a primary deficiency in P5CS during hyperammonemia.

The main treatment of PA is dietary restriction of propiogenic amino acids to avoid the formation of propionic acid. As a result, their levels in the stable state are low. However, it is well known that excessively low concentrations of Val and especially Ile are related to acrodermatitis enteropathica-like lesions (Oztürk 2008). For this reason, supplementation is often required. The effect of excessive restriction of these amino acids goes beyond that. We have previously demonstrated that low Val levels can also be related to the chronic anemia that some of these patients suffer (Stanescu et al. 2021). Low Val and Ile levels in relation with high Leu concentrations is the usual scenario in PA (Molema et al. 2019) and has recently been related to various deleterious mechanisms in maple syrup urine disease patients (Strauss et al. 2020). We now wonder if dietary restriction for Ile and Val could hinder Gln formation in muscle and other organs (Neinast et al. 2019) favoring a chronic deficit in TCA and energy cell production that could explain some of the acute and chronic complications observed in these patients. We certainly recommend aiming for the highest possible natural protein intake, and only restricting for short periods of time after a decompensation event. During these episodes, supplementation with Cit and Arg might help overcome some of the amino acid disbalance that we have observed and enhance ammonia elimination.

Our study has several limitations. The small sample of patients makes difficult finding differences related to age or sex. Although the inclusion of multiple tissues offers an interesting physiopathologic model, it does not prove the full picture. Many other tissues can contribute to BCAAs oxidation besides muscle or liver, such as brown fat, cardiac muscle, or kidney and, interestingly, pancreas supplies 20% of its TCA carbon from BCAAs (Neinast et al. 2019).

The plasma profile we observe points to an important amino acid exchange among different metabolic pathways and organs, playing a central role in the pathogenesis of this disease. Therapies focused solely in one of them will be insufficient to solve them all, and that could explain why liver transplanted patients still suffer certain complications (Pillai et al. 2019). However, enhancing propionyl-CoA carboxylase function in the body and, also very importantly, allowing for a greater natural protein intake are good reasons to consider it in those patients in which other approaches fail. Our results offer a new physiopathological model in PA, connecting the deficient anaplerosis to urea cycle dysfunction, with immediate clinical implications.

## Conclusions

Propionic acidemia affects the metabolism of 4 of the 9 essential amino acids. It is not surprising to learn that this enzymatic dysfunction has implications in multiple and fundamental metabolic pathways, both directly and indirectly. Our findings show a lack of production of glutamine (Gln) and alanine (Ala) that can at the same time be a sign and a reason for reduced anaplerosis in PA and could help explain some of the complications observed. Circulating Gln depends on skeletal muscle production, but its reduction affects enterocyte, kidney and liver urea cycle-related pathways. The inter-organ amino acid interchange taking place in this disease is necessary to better understand its physiopathology in the search for better therapeutic options for our patients.

## Data Availability

All data generated or analyzed during this study are included in this published article. Upon request authors will send relevant documentation or data in order to verify the validity of the results presented.
